# The Role of Next-Generation Sequencing (NGS) in the Management of Tuberculosis: Practical Review for Implementation in Routine

**DOI:** 10.3390/pathogens12080978

**Published:** 2023-07-26

**Authors:** Marion Beviere, Sophie Reissier, Malo Penven, Loren Dejoies, François Guerin, Vincent Cattoir, Caroline Piau

**Affiliations:** 1 Service de Bactériologie-Hygiène Hospitalière, CHU de Rennes, F-35043 Rennes, France; 2 Inserm U1230, Université de Rennes 1, F-35043 Rennes, France; 3 CNR de La Résistance Aux Antibiotiques (Laboratoire Associé ‘Entérocoques’), F-35033 Rennes, France

**Keywords:** tuberculosis, next-generation sequencing, antibiotic resistance

## Abstract

Next-generation sequencing (NGS) has modernized the field of tuberculosis (TB) research by enabling high-throughput sequencing of the entire genome of *Mycobacterium tuberculosis* (MTB), which is the causative agent of TB. NGS has provided insights into the genetic diversity of MTB, which are crucial for understanding the evolution and transmission of the disease, and it has facilitated the identification of drug-resistant strains, enabling rapid and accurate tailoring of treatment. However, the high cost and the technical complexities of NGS currently limit its widespread use in clinical settings. International recommendations are thus necessary to facilitate the interpretation of polymorphisms, and an experimental approach is still necessary to correlate them to phenotypic data. This review aims to present a comparative, step-by-step, and up-to-date review of the techniques available for the implementation of this approach in routine laboratory workflow. Ongoing research on NGS for TB holds promise for improving our understanding of the disease and for developing more efficacious treatments.

## 1. Introduction

Tuberculosis (TB) results from infection by the *Mycobacterium tuberculosis* complex, which is one of the oldest diseases known to affect humans and a major cause of death worldwide. In 2021, the WHO reported that 10.6 million people had contracted TB and 1.6 million deaths had been attributed to it, which ranked it thirteenth among the chief causes of mortality worldwide, and the second among infectious (after COVID-19) [1]. The emergence of resistance to first- and second-line anti-TB drugs represents a real concern in terms of public health. Beyond the challenge of the choice of therapeutic, such resistance entails long and costly treatments, delays in antibiotic tailoring, and the requirement of appropriate sites and protective equipment. The development of rapid diagnostic tests and the determination of drug susceptibility are essential to provide appropriate anti-TB patient care as early as possible.

The advent of molecular diagnostic tools has made it possible to overcome culture delays and the need for adequate infrastructures to achieve this. Firstly, with targeted PCR, and then with approaches incorporating resistance genes to first- and second-line anti-TB, it is with next-generation sequencing (NGS) that the future of molecular diagnostics for tuberculosis now seems to be taking shape. By visualizing all of the molecular “hotspots” of resistance, as well as the mutations not yet systematically associated with phenotypic resistance, NGS provides answers to many individual and collective questions, as well as opening up new avenues of research into TB.

## 2. Mechanisms of Resistance to Anti-TB Drugs

Acquired resistance of the *M. tuberculosis* complex to anti-TB drugs is always chromosomal, and it results from mutations in genes that generally code for the targets of these antibiotics. Advances in molecular biology have enabled recent descriptions of a significant number of mutations, but they are not systematically related to the phenotypic resistances described. Further studies are therefore needed to complete the data. The WHO provides an online free catalogue of the mutations described in the *M. tuberculosis* complex and their associations with drug resistance [2]. The catalogue provides a reference standard for the interpretation of mutations conferring resistance to all first-line drugs and a variety of second-line drugs. The report summarizes the analysis of over 38,000 isolates with matched data on whole-genome sequencing (WGS) and phenotypic drug susceptibility testing from over 40 countries for 13 anti-TB drugs. It lists over 17,000 mutations, their frequency, and their possible association with resistance, and it includes the methods used, the mutations identified, and summaries of important findings for each drug. To standardize the analysis, only sequencing performed with the Illumina^®^ technique is included. All of this data are complemented by results from a reference phenotypic susceptibility test, as far as possible performed with an approved method. For each antibiotic, mutations are classified according to their impact on susceptibility to antibiotics: Resistance Associated Mutation (Assoc w R), Mutations Not Associated with Resistance (Not assoc w R), and, finally, mutations for which the impact is uncertain (Uncertain Significance). The “interim” category consists of data not reviewed and approved by WHO techniques, or with uncertainty for some of the associations observed, and it could therefore change categories in the future. For each antibiotic, sensitivity (Se), specificity (Sp), positive predictive value (PPV), likelihood ratios (LR), and odds ratios (OR) for all mutations are also indicated. This enables the performance of confidence-graded mutations for predicting phenotypic drug susceptibility to be estimated. Performance for some drugs may appear suboptimal in this data, but the prevalence of resistance to these drugs in the population should be taken into account. Finally, it should be noted that the main mutations involved in resistance are limited, which has enabled the implementation of targeted molecular biology tests [2].

## 3. Molecular Detection of Resistance Mutation in MTB: PCR versus NGS

Targeted PCR techniques have been optimized in line with the evolution of the disease, whether in terms of epidemiology or resistance worldwide. It is well known that early detection of resistance to first-line treatments is essential for the implementation of appropriate antibiotic therapy and to prevent the emergence of resistance. The detection of rifampicin resistance mutations in the *rpo*B gene is therefore a priority. Shortly after this, multiplex PCRs enabled broader studies on genotypic second-line anti-TB drugs, such as fluoroquinolones and/or aminoglycosides. This has enabled faster detection of PreXDR and XDR-TB, albeit with certain limitations. Indeed, only previously defined mutations on the target genes are detected. Although these tests target the most frequently found mutations in cases of multi-resistant TB, other resistance mutations, sometimes targeting new genes, are emerging, and the sensitivity of these tests, although effective, can be inadequate to detect resistance. Under-detection of rare mutations can theoretically expose them to the emergence of multidrug-resistant strains. Table 1 lists the main PCR techniques for detecting resistance according to the anti-TB drugs targeted.

**Figure 1:**
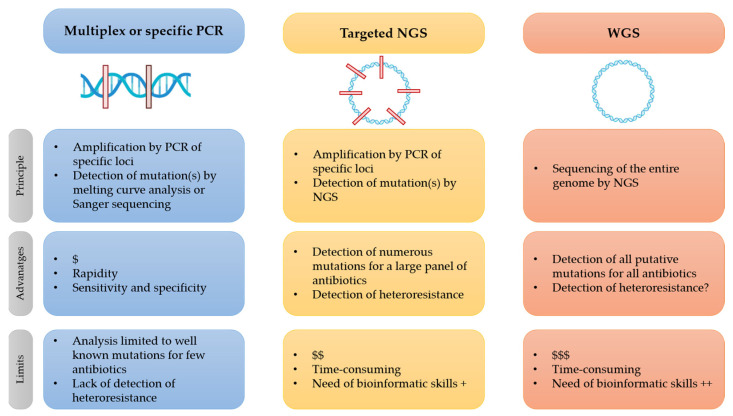
Advantages and limits of molecular techniques for detection of resistance mutations in MTB.

The WGS approach for the detection of resistance mutation offers new possibilities to study resistance mutations, of course, but also to study the genotyping strain that can control epidemic diffusion. Figure 1 shows the principles, advantages, and limits of “classical” targeted PCR for the detection of resistance mutations versus the high-throughput sequencing method, targeted NGS, and WGS.

## 4. Prerequisites for the Molecular Analysis of the *M. tuberculosis* Complex Genome: Principles, Particularities, and Limitations of DNA Inactivation and Extraction

### 4.1. NGS Principles

NGS is a technique for the high-throughput sequencing of several genes simultaneously for subsequent comparison with reference sequence libraries. NGS applications include target NGS and whole-genome sequencing (WGS). The former enables the sequencing of certain parts of the genome, targeting genes of interest, and it is therefore useful when studying acquired resistance in bacteria. In the second case, the entire genome is sequenced, making it extremely useful for research and epidemiological monitoring of tuberculosis, to take just two examples. Both techniques can be performed on the same sequencer, using DNA extracts from bacterial cultures or clinical samples. In the latter case, it is possible to obtain good results with targeted NGS, but the presence of large quantities of human DNA greatly reduces sensitivity. These high-throughput sequencing techniques can now be used routinely, but a number of factors limit their use to reference laboratories. Firstly, the cost of the equipment and the analyses remains high, even if the trend is towards democratization, but, above all, their use requires skilled and qualified personnel, both technically and biologically/bioinformatically. 

Unlike other molecular biology techniques, NGS enables the detection of heteroresistance, which is defined as resistance to certain antibiotics expressed by a subset of a microbial population that is generally considered to be sensitive to these antibiotics in in vitro sensitivity tests. Interpretation of the susceptibility of these sub-populations and their clinical impact can be difficult, but this phenomenon was already identified by conventional phenotypic methods, such as the determination of antibiotic susceptibility by the proportion method [13]. 

When talking about NGS, there are several parameters to consider. Firstly, an important concept to take into account is the important concept of reading depth, which is the average number of reads per base at a given position. The greater the depth, the greater the number of overlapping reads that can be assembled, and the greater the fraction of the genome covered. Moreover, the number of sequences observed at a given position in the genome is a quality criterion [14]. When studying the bacterial genome, it is therefore important that the NGS technique used achieves significant depth (ideally 10×) in order to highlight the presence of minority variants that may carry resistance mutations and risk causing therapeutic failures.

In terms of method, a number of successive steps are required. First, the extracted DNA must be fragmented and, if necessary, amplified by PCR, and then a library must be prepared by attaching adapters and indexes to the fragments, which are then attached to a surface for sequencing for the individual fragments to be identified. This library is then amplified to form clusters, which are then sequenced. Different sequencing techniques can be used and a number of parameters are variable, such as the initial quantity of DNA, the preparation of the libraries with (in particular) a significant difference in the size of the DNA fragments to be sequenced, and then the sequencing technique itself as well as the detection system incorporated into the system. They can thus be classified as second- and third-generation. The second-generation sequencing technique consists of sequencing by synthesis, which generates millions of reads of small fragments of around a hundred bases (called, simply, ‘reads’) with very few read errors. One of the problems with this technique is that the read is in a highly fragmented form, making it difficult to reconstruct the genome, particularly because of repeated regions to detect certain variants. This technique requires amplification by GC-rich fragments, which are generally less well amplified and therefore under-represented. This is why a new generation of NGS called “third generation” has been developed, which is based on a technique of single-molecule sequencing (SMS), generating much longer reads of tens of kilobases but in just a few thousand copies. This technique gives a much lower reading accuracy than that of second-generation techniques, but read errors are randomly distributed. Interpretation therefore requires an algorithm to help correct these reading errors. This technique does not require an upstream PCR amplification step. Third-generation sequencing techniques use the SMRT (single molecule real time) sequencing technique, in which each new base introduced during DNA polymerase synthesis is detected by fluorescent polymerase or by a change in ionic current when using nanopores [15].

**Figure 2:**
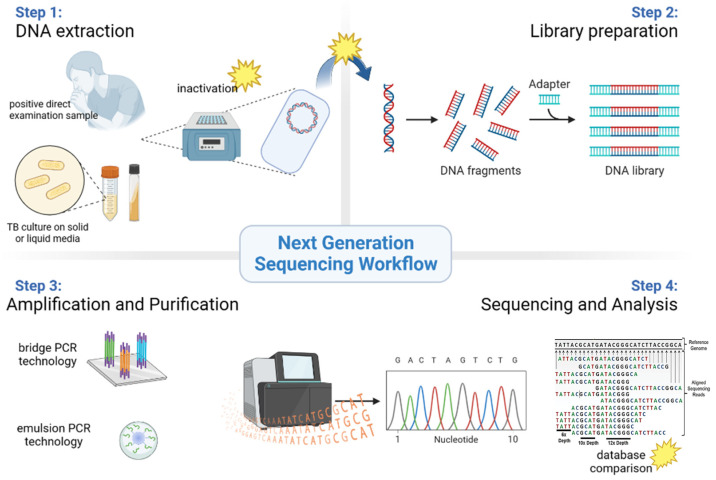
NGS workflow from sample to sequences analysis; stars symbolize critical steps of the process.

Figure 2 describes each critical step in the NGS process, which we will detail further.

### 4.2. Inactivation 

Molecular biology on MTB complex strains is challenging because of their intrinsic bacterial particularities. Inactivating the bacteria while preserving the integrity of the DNA can be problematic. Several thermal and chemical techniques have been described. The classically established standard protocol consists of inactivation by heating at 80 °C for 20 min. Nevertheless, studies have contradicted one another regarding the efficacy of this protocol. Doig et al. observed no colonies after prolonged culture on specific Lowenstein–Jensen medium (20 weeks) and MB/BacT bottles (12 weeks) after immersion of strains in a water bath, thereby providing proof of the efficacy of this technique [16]. Conversely, in their study, Somerville et al. demonstrated the persistence of viable mycobacteria using this same inactivation protocol combined with the use of lysozyme and proteinase K: 77% (27/35) of the Lowenstein–Jensen cultures carried out after inactivation were positive within a median of 17.5 days, and 20% (7/35) of the BACTEC vials were positive within a median of 35 days of incubation [17]. In this study, it was not specified whether the heating was carried out in a dry bath or by immersion. Similar results were described in the study by Bemer-Melchior et al. using a water bath at 80 °C [18]. More recently, a study published by Billard-Pomares et al. also showed the presence of 40% positive cultures after using the dry-bath thermal inactivation protocol [19]. The 39 cultures were carried out on Coletsos solid medium that was incubated for 90 days and MGIT liquid medium that was incubated for 42 days. 

Another important point to consider is the preservation of bacterial DNA integrity after heating. Bemer-Melchior et al., using electrophoretic migration of DNA fragments on a 1% agarose gel, demonstrated the denaturing effect of thermal inactivation at 80 °C for 20 min [18]. This finding was contradicted by Billard-Pomares et al., who showed an equivalence in the results obtained on whole-genome sequencing (WGS) with or without previous thermal inactivation (with the protocol consisting of heating in a dry bath at 80 °C for 15 min in this study) [19]. In view of these discrepancies, it seemed relevant to look into other techniques for inactivating the *M. tuberculosis* complex to find the best compromise between the safety of the technique and the quality of the DNA obtained. Bemer-Melchior et al. suggested a modified thermal protocol consisting of heating strains in a water bath at 100 °C for only 5 min, and they obtained good results both in terms of the inactivation and the preservation of genome integrity [18]. Other tests have been carried out to obtain an effective inactivation of the M. tuberculosis complex by chemical processes, and one example consists of incubating colonies in the presence of chloroform for 20 min and then 70% ethanol for 30 min, and this protocol, alone or in combination with a thermal inactivation technique, has shown excellent results. No post-treatment samples, whether on liquid or solid medium, showed positive cultures within 42 and 90 days, respectively, and WGS applications were successful [19].

### 4.3. DNA Extraction 

DNA extraction of the *M. tuberculosis* complex can be carried out from an inactivated strain, either from a solid medium culture or from the pellet of a liquid medium, but it can also be performed directly from a clinical sample when the smear test is positive for acid-fast coloration, or when DNA is detected by specific PCR. However, several parameters make DNA extraction delicate. First, the first-line diagnosis of pulmonary TB is made on sputum; this type of sample can contain PCR inhibitors and exogenous DNA, and it requires a first liquefaction step leading to a change in pH. In addition, mycobacteria have a complex cell wall containing many polysaccharides, thus making cell lysis difficult and potentially affecting downstream analyses. However, efficient extraction is essential for the performance of molecular biology techniques, including NGS. There is no gold standard, but many extraction protocols can be used to extract the DNA of the *M. tuberculosis* complex, from manual “home-made” techniques to automated extractions with commercial kits with variable performances. Results can fluctuate depending on the matrix (sample or culture) and on the molecular biology technique used (targeted PCR, NGS, etc.). To achieve a satisfactory extraction yield, several steps may be necessary, with a primary stage for the liquefaction of the sample and/or cell lysis [20].

Table 2 provides a summary of some examples of commercial and non-commercial manual extraction methods based on a recent literature review. The starting matrix and the molecular biology techniques applied to the extracts are variable and can be difficult to compare. Different parameters need to be taken into account: extraction yield, purity of the extract, and technical parameters, such as the complexity of the technique, the overall time of extraction, the time required, the number of steps performed, the equipment required, and the cost of the reagents. Many protocols have been established for PCR tests, but yield and urity are very often insufficient for WGS prerequisites [21].

Recently, more and more automated DNA extraction kits have been commercialized. Colman et al. compared these different techniques using different criteria:

– Organizational: volume and flow capacities and flexibility, random access, extraction time, and integration of the step of cell lysis in the technique.
– Techniques: tests already performed on the *M. tuberculosis* complex and on sputum, the proportions of the automated system, and reagent storage.
– Economic: the cost of the platform and the reagents.
– Quality certification: the presence or absence of CE-IVD or FDA marking.

Unfortunately, extraction efficacy was not compared because most of the data come directly from suppliers and not from independent experimenters. Following this comparison, 18 potential instruments/kits were selected. Of these, the sixth highest-scoring combinations are listed in order in Table 3 [20].

## 5. Detection of Resistance Genes in the *M. tuberculosis* Complex by NGS

### 5.1. NGS Techniques: Principle and Applications

NGS is based on the high-throughput sequencing of thousands of genes simultaneously, and the resulting sequences are then compared to worldwide reference databases. Among the NGS applications, a distinction should be made between “Target NGS” and WGS. Unlike other molecular biology techniques, NGS enables the detection of heteroresistance, which is defined as resistance to certain antibiotics expressed by a subset of microbial populations that are generally considered to be susceptible in vitro [13]. 

NGS requires a first step of fragmentation of the extracted DNA and sometimes an amplification step using PCR. The next step is to prepare a library by affixing adapters and indexes to these fragments. Adapters are used for fixing on a surface for sequencing and also for the identification of different fragments. This library can, especially in Illumina^®^ techniques, be amplified to form clusters that are subsequently sequenced. 

Several parameters are then variable: initial quantity of DNA, library preparation with a significant difference in DNA fragment sizes, and the sequencing technique itself, as well as the detection system that is incorporated into the system. These differences divide equipment into second- and third-generation sequencing. 

The second-generation sequencing technique consists of sequencing by synthesis, which generates millions of readings of small fragments of about a hundred bases with very few reading errors. One of the main pitfalls of this technique is the excessive fragmentation of the sequence readings, which makes it difficult to collate, especially for repeat regions. This technique does not always enable the detection of certain variants. In addition, it requires PCR amplification steps, but GC-rich fragments are generally less amplified and are therefore underrepresented. 

The third-generation technique uses SMS (single molecule sequencing), generating longer reads (tens of kilobases) but in only a few thousand copies. This technique provides very reduced reading precision compared to second-generation techniques, but the reading errors are distributed randomly. Interpretation requires an algorithm to correct these reading errors. It is worth noting that this technique does not require an upstream PCR amplification step.

To evaluate the quality of the sequences thus obtained, the important notion of the reading depth should be taken into account. This is the average number of readings per base at a given position. The greater the depth, the more overlapping readings can be collated, and, thus, the larger the fraction of the genome covered. In addition, the number of sequences observed at a specific position in the genome is a quality criterion [14]. It is important during the characterization of the bacterial genome for the NGS technique to enable a substantial depth (a minimum of 50) in order to highlight the presence of minority variants that could carry resistance mutations and could risk causing treatment failures.

Table 4 presents examples of commercialized kits, their respective compatibilities with the sequencers, and their possible applications.

In a study led by the WHO in 2018, different automated systems of high-throughput sequencing were compared, and their advantages and disadvantages are shown in Table 5. Illumina^®^ technology is currently the most widely used technology worldwide. The Qiagen^®^ GeneReader System was not included in this comparison because of a lack of independent data in the literature.

### 5.2. Databases and Interpretation

After interpretable sequences are obtained, they are entered into the databases in order to detect any mutations and to obtain interpretative results by comparison with the published data, which, at the same time, enables the identification of the organism and the detection of potential mutations in the genes of interest. Hendriksen et al. [28] listed more than 47 databases in a recent review. Some are open access, others make charges, some of them specialize in mycobacteria, and others are more general. The data source implementing the system differs from one to the next, as does the number of genes included and, therefore, the number of anti-TB drugs covered. 

Table 6, which is based on the recent data from the literature, presents a non-exhaustive list of the different databases that can be used for sequence analyses.

## 6. Discussion 

TB is still a global public health issue today. It is currently the second highest cause of death from an infectious disease, after COVID-19. For several years, high throughput sequencing has been progressively implemented in laboratories, including in bacteriology. Its impact is now being evaluated in the diagnosis of TB and the production of genotypic AST. In particular, NGS has overcome the delay in culture of the *M. tuberculosis* complex. 

Today, many approaches, commercial or other, are available, but with very unequal performances and an absence of a consensus on the key steps in the process. First, the efficacy of inactivation protocols, which is essential for the protection of personnel and premises, is debated in the literature. It is therefore important to carry out internal tests to ensure safety in each laboratory concerned. It is also necessary to overcome the difficulties of extracting bacterial DNA. For this, more or less efficient extraction methods are available, and they need to be evaluated in order to combine simplicity, robustness, and yield. Future challenges are to optimize existing methods in order to improve the signal, either by enriching the DNA amount or by eliminating the background noise from clinical samples. Thus, some papers have recently proposed DNA enrichment on clinical specimens by capture methods––either by microfluidics-based cell capture [39,40] or by magnetic bead [41]. Capture methods may be promising for enrichment in clinical sputum samples, which could facilitate culture-free MTB WGS. Other researchers propose to find low abundance sequences by hybridization (FLASH) to enrich targeted sequences [42], or propose a pipeline that can help to clean contaminant reads from sputum samples and/or detect mixed infections [42,43,44,45]. 

After making the critical choices of the capture method to be used, a sequencing approach then needs to be chosen, too, taking into account its cost effectiveness and performance. While the Illumina^®^ method remains the most widely used technique worldwide, others are available, particularly with the arrival of third-generation sequencers. Finally, many databases are available; some are specific to mycobacteria, but a large number of them are neither updated regularly nor consolidated by phenotypic/genotypic comparison data. All of this shows the need to publish guidelines for the application of NGS in the diagnosis of TB and the detection of resistance mutations on a large scale. In the long term, the objective is to democratize the use of NGS by way of simple, fast, and accessible tools, extending to the Point-Of-Care Test (POCT), as close as possible to patients, with support and immediate therapeutic adaptation as well as the possibility of rapidly breaking the cycles of TB transmission. The Deeplex^®^ project [36], supported by the WHO, contributes to this by proposing an integrated approach and a readable representation of the mutations of the genes of interest. In addition, a global objective can be derived from this approach to the sequencing of the *M. tuberculosis* complex. On the one hand, this could enable data on the phylogeny of the strains to be obtained and their circulation in the human population to be studied, and it could also provide a better understanding of the bacterial genome, which would allow in silico therapeutic targets for candidate drugs to be designed. Finally, other pathologies where the impact is more limited in terms of the number of cases but where the consequences of that impact remain serious, such as leprosy or Buruli ulcer, could benefit from the same approach, as could the diagnosis of opportunistic infections with atypical mycobacteria.

## 7. Conclusions

Diagnosis of tuberculosis is challenging for routine laboratories, and it has been recently revolutionized by using molecular methods. However, a characterization of the resistance profile of MTB often needs a cultural step, which can take anywhere from days to several weeks. It is important to keep in mind that the crucial objective is always to treat the disease well and fast in order to prevent additional drug resistance mutations during the empirical treatment of TB. The conventional PCR approach could screen hot-spot mutations for a great majority of patients, but the WGS route is a very promising option for free treatment from culture delay, especially for resistant strains. Implementation of this approach in routine practice needs to be preceded by a reflection on mycobacterial intrinsic particularities, especially for inactivation and DNA extraction, in order to guarantee good sequencing performance. Finally, the analysis method choice is fundamental, too, and the WHO’s recommendations are also valuable to the process of harmonizing interpretations of known *but also putative* mutations. WGS is now a well-described method on culture samples, but it could also be used for direct examination of positive samples, which are sometimes limited by commensal bacteria and host DNA. Optimization by DNA enrichment methods and the harmonization of existing techniques are now both required to allow a rapid and reliable diagnosis of a resistance TB profile. 

## Figures and Tables

**Table 1:** Comparison of the main PCR techniques in detection of resistance mutations to first- and second-line anti-TB drugs.

Device (Manufacturer)	CE-IVD	Validated Samples	Techniques	Time (Per Sample)	Target Genes		Ref.
**PCR techniques for the detection of rifampicin resistance mutations**							
**Xpert^®^ MTB/RIF Ultra (Cepheid^®^)**	Yes	Sputum	Closed system, extraction, and then nested semi-quantitative PCR. Detection limit: 11.8 CFU/mL. Manual technical time < 1 min.	<80 min	*rpoB* (+IS*6110* and IS*1081*)		[3]
**Truenat™ MTB-RIF Dx (Molbio^®^)**	Yes	Sputum	Extraction not included. Quantitative real-time PCR. Detection limit: 200 CFU/mL.	1 h	*rpoB*		[4]
**PCR techniques for the detection of rifampicin and isionazid resistance mutations**							
**MDR-TB** **(BD MAX™)**	Yes	Sputum	Fully automated system with extraction cassette and semi-quantitative real-time PCR. Detection limit: 6 CFU/mL. Manual technical time < 1.5 min.	<4 h	*rpoB* *inhA* *katG*		[5]
**MDR/MTB ELITe MGB^®^ (ELITechGroup)**	Yes	Sputum, urine, BAL, bronchial aspirate cavitary fluid, gastric aspirate, tissue/biopsy	Fully automated system with extraction cassette and semi-quantitative real-time PCR. Detection limit: 6 CFU/mL. Manual technical time < 2 min.	<3 h	*rpoB* *inhA* *katG*		[6]
**FluoroType^®^ MTBDR VER 2.0** **(BRUKER)**	Yes	Sputum and culture	Extraction not included. Real-time PCR amplification and automated detection in a closed system. Detection limit: 20 CFU/mL.	2,5 h	*rpoB* *inhA* *katG*		[7]
**GenoType^®^ MTBDRplus VER 2.0** **(BRUKER)**	Yes	Lung samples and cultures	Extraction not included. Amplification by PCR and hybridization on strips and enzymatic revelation. Detection limit: 160 CFU/mL.	5 h	*rpoB* *inhA* *katG*		[8]
**MTB MDR Real-TM** **(Sacace)**	No	ND	Extraction not included. Qualitative real-time PCR.	ND	*rpoB* *inhA* *katG*		[9]
**Anyplex™II MTB/MDR** **(Seegene)**	Yes	Sputum, BAL, culture and other fresh tissues	Extraction. Real-time PCR.	3 h	*rpoB* *inhA* *katG*		[10]
**PCR techniques for the detection of second-line resistance mutations**							
**Xpert^®^ MTB/XDR** **(Cepheid^®^)**	Yes	Sputum	Closed system, extraction, and nested semi-quantitative PCR. Detection limit: 136 CFU/mL. Manual technical time < 1 min.	<90 min	*inhA* *katG* *fabG1* *ahpC*	*gyrA* *gyrB* *rrs* *eis*	[11]
**GenoType^®^ MTBDRsl VER 1.0** **(BRUKER)**	ND	Lung samples and cultures	Extraction not included. Amplification by PCR and hybridization on strips and enzymatic revelation.	5 h	*gyrA* *rrs* *embB*		[8]
**GenoType^®^ MTBDRsl VER 2.0** **(BRUKER)**	Yes	Lung samples and cultures (v2 validated even on negative direct examination)	Extraction not included. Amplification by PCR and hybridization on strips and enzymatic revelation. Detection limit: 150 CFU/mL.	5 h	*gyrA* *gyrB* *rrs* *eis*		[8]
**Anyplex™** **II MTB/XDR** **(Seegene)**	Yes	Sputum, BAL, culture, and other fresh tissues	Extraction not included. Real-time PCR.	3 h	*gyrA* *rrs* *eis*		[12]
**Anyplex™** **II MTB/MDR/XDR** **(Seegene)**	Yes	Sputum, BAL, culture, and other fresh tissues	Extraction not included. Real-time PCR.	3 h	*rpoB* *inhA* *katG*	*gyrA* *rrs* *eis*	[12]

**Table 2:** Comparison of different manual extraction techniques for M.tuberculosis complex DNA. BAL Bronchoalveolar lavage, HM home-made technique; C: commercial technique; enriched Sputum: Sputum enriched with M. tuberculosis ATCC 27294; WGS: Whole Genome Sequencing nd: not determined.

Principle	Type of Technique	Sample	Applications	Extraction Protocol	Performance	Reference
**Thermolysis:** A total of three successive cycles of boiling and freezing at −20 °C.	HM	Solid medium culture	Identification by PCR-IS6110 and genotyping by spoligotyping	2 h	No prior inactivation required. Good performance for identification and genotyping.	[22]
**In-house chelating resin:** 5% Chelex-100 + Tris + EDTA boiled with the sample pre-treated with phosphate buffer.	HM	Sputum, BAL, biopsy.	Multiplex PCR for the amplification of antibiotic resistance target genes	<1 h	Extraction yield, 5.2 ng/µL, requires a large sample volume to perform multiplex PCR. Presence of PCR inhibitors in the resin. Performance in the detection of various genes.	[23]
**InstaGene Matrix:** Chelating resin: 6% Chelex-100, incubated at 56 °C then 100 °C with the sample.	C	Sputum, BAL, biopsy.	Multiplex PCR for the amplification of antibiotic resistance target genes	<1 h	Extraction yield, 4.5 ng/µL, requires a large sample volume for multiplex PCR. Presence of PCR inhibitors in the resin. Variable gene detection performance but slightly better than in-house technique.	[23]
**Chemical technique with CTAB:** Cationic Detergent Cetyltrimethylammonium Bromide. Protocol in several incubation steps with different reagents: 1-Lysozyme 2-Proteinase K + SDS 3-CTAB 4-Chloroform isoamyl alcohol 5-Isopropanolol	HM	solid medium culture	Identification by PCR-IS6110 and genotyping by spoligotyping	30 h	Good performance in identification and genotyping.	[22]
		Sputum	Real-time sequencing	7 h	Lower DNA concentration and extraction yield (1.88 ng/µL) compared to commercial kit (PrimeXtract kit). Variable purity but slightly higher than the commercial kit and low cost. Possible application in real-time sequencing but with the use of a large sample volume.	[24]
		liquid medium culture	Identification by PCR-IS6110	nd	Good performance in performing PCR-IS6110 with an extraction yield of 31.2 ng/µL.	[25]
**Chemical and enzymatic:** Incubation with: 1-Chloroform, methanol 2-Lysozyme 3-SDS and proteinase K 4-Purification with chloroform, phenol and ethanol	HM	Liquid medium culture	Identification by PCR-IS6110	20 h	Equivalent performance to the chemical technique with CTAB for the IS6110 PCR with a better extraction yield (174 ng/µL).	[25]
**Chemical, enzymatic + Stirring balls:** Addition of a step compared to the previous technique: Mechanical disruption using tubes containing zirconium beads in buffer (SDS-EDTA-proteinase K).	HM	Liquid medium culture	Identification by PCR-IS6110	>24 h	Good performance in PCR-IS6110 with better extraction yield (240 ng/µL) compared to the CTAB method, but DNA splitting is deleterious for use in WGS.	[25]
**Tris-EDTA Lysis Buffer:** Boiled.	HM	Enriched Sputum	Quantitative PCR-IS6110	<1 h	Good performance for PCR-IS6110 but low extraction efficacy. The yield can be increased by using lysis tubes containing glass beads into which the buffer is introduced or by the use of specific reagents (PrepMan™). Very low cost but more costly when using lysis tubes or specific reagents.	[26]
**2% SDS detergent and 10% Triton X-100:** Boiled.	HM	Enriched Sputum	Quantitative PCR-IS6110	nd	PCR inhibition can be explained by SDS inhibition of Taq polymerase.	[26]
**QIAGEN QIAamp DNA mini kit:** Column extraction.	C	Enriched Sputum	Quantitative PCR-IS6110	3 h	Enzyme pre-treatment with lysozyme and proteinase K. Good performance for PCR-IS6110 but low extraction efficacy. High cost.	[26]
		Liquid medium culture	WGS	nd	Inactivation required. Low extraction yield, and low purity, not suitable for use in WGS (<0.2 ng/µL)	[21]
**QuickGene DNA Kit:** For use with the semi-automatic QuickGene-Mini80 system	C	Liquid medium culture	WGS	nd	Inactivation required. Better extraction efficacy than Qiagen QIAmp mini but insufficient for use in WGS (±0.3 ng/µL).	[21]
**Precipitation with ethanol + Pretreatment with MolYsis kit or saline solution:** **Multi-step protocol.** 1-Purification with MolYsis kit reagent or saline solution. 2-Cell lysis with bead lysis tube. 3-Precipitation with ethanol. 4-Washing with magnetic beads.	HM	Liquid medium culture	WGS	>3 h	Numerous manual steps. Extraction yield, better than QIAGEN QIAamp DNA mini and QuickGene DNA kits. Possible use for WGS (±2 ng/µL). Efficacy of the MolYsis kit to eliminate human DNA but high cost. Slightly lower performance for saline washing but lower cost.	[21]
**PrimeXtract kit:** Extraction on columns with ready-to-use lysing and washing solutions.	C	Sputum	Real time sequencing	<1 h	Good extraction yield (5.93 ng/µL), low purity, and insufficient quantity of DNA to perform sequencing directly on sputum. Additional decontamination and purification steps are required. High cost.	[24]

**Table 3:** Pros and cons of the five highest-scoring combinations of automatic extraction machines and extraction kits from the study by Colman et al. [20].

Techniques	Advantages	Disavantages
**Nextractor** (Genolution) with B/NTMExtraction kit	- High throughput: 1 to 48 samples. - Extraction time = 10 min. - Low cost of machine: $12,000. - Lowest cost per sample: $2. - Specific kit for *M. tuberculosis* complex.	- Low volume: 0.25 mL.
**Trueprep Auto** (MolBio) with MycobacterialDNA Isolation kit	- Extraction time = 20 min. - Lowest cost of the machine: $3,000. - Low cost per sample: $3. - Battery-powered. - Small size.	- Low throughput: only 1 sample at a time.
**Arrow** (DiaSorin) with BUGS’n BEAD kit	- Throughput: 1 to 12 samples. - Extraction time = 30 min. - Cost of machine: $16,415. - Cost per sample: $6.22.	- Low volume: 0.2 or 0.5 mL.
**AnaPrep12 and AnaPrep24** (Biochain) both combined with AnaPrep TB DNAExtraction kit	- High throughput: 1 to 12 or 24 samples. - Cost of machine: $19,500 (AnaPrep12). - Cost per sample: $4.52. - Largest volume: 0.1-1.2 mL. - Specific kit for *M. tuberculosis* complex.	- Extraction time = 60 min - Large size, bulky.
**SimplePrep X8 instrument -PureLyse** (Claremont bio) with PureLyseBacterialgDNA Extraction kit	- Throughput: 1 to 8 samples. - Extraction time = 6 min, fastest. - Cost of machine: $25,000. - Small size.	- Higher cost per sample = $13. - Low volume: 0.5 mL.

**Table 4:** Comparison of library preparation kits produced by WHO and ONT (Oxford Nanopore Technologies) [27].

Library Prep Kit	Sequencer Compatibility	Applications	Amount of DNA Required
**Nextera XT**	All the Illumina	WGS and target NGS	1 ng
**Nextera DNA Flex**	All the Illumina	WGS and target NGS	1–500 ng
**AmpliSeq**	All the Illumina	Target NGS	1–100 ng
**Ion Xpress Plus Fragment**	PGM and S5 Ion Torrent	WGS and target NGS	100 ng
**MuSeek**	PGM and Proton	WGS and target NGS	100 ng
**Rapid Sequencing Kit**	All the ONT	WGS and target NGS	400 ng
**Ligation Sequencing Kit 1D**	All the ONT	WGS and target NGS	1000 ng
**Low Input by PCR Sequencing**	All the ONT	WGS and target NGS	<100 ng
**1D^2^ Sequencing**	All the ONT	WGS and target NGS	1000 ng

**Table 5:** Comparison of sequencing machines produced by the WHO [27]. Fluo: Fluorescence; PR: Semiconductor detecting pH change by proton release; IC: Change in ionic current; SMRT (single molecule real time technology).

Sequencer (Manufacturer)	Detection Technique	Data (Gb)	Maximum Read Length (bp)	Sequencing Time	Estimated Cost of the Sequencer (USD)	Advantages	Disadvantages
**Third-generation** **sequencing by synthesis. Bridge PCR.**							
iSeq (Illumina)	Fluo	0.3−1.2	2 × 150	9−17.5 h	19,900	Price Sequencing time	Fragment size Low throughput
MiniSeq (Illumina)	Fluo	1.7−7.5	2 × 150	4−24 h	50,000	Price Sequencing time	Fragment size Low throughput
MiSeq (Illumina)	Fluo	0.3−15	2 × 300	4−55 h	100,000	Fragment size	Sequencing time
NextSeq (Illumina)	Fluo	10−120	2 × 150	12−30 h	250,000	Throughput	Fragment size Sequencing time
HiSeq -2500 (Illumina)	Fluo	10−1000	2 × 150	<3 h	650,000	Throughput Reading accuracy	Fragment size Sequencing time Price
Nova Seq (5000/6000) (Illumina)	Fluo	2000−6000	2 × 150	16−44 h	850,000−950,000	Sequencing time Reading accuracy	Fragments size Sequencing time Price
PGM (Thermo Fisher Scientific)	PR	0.08−2	400	3−10 h	80,000	Sequencing time Read length	Throughput Homopolymers
S5 (Thermo Fisher Scientific)	PR	0.6−15	400	>19 h	60,000	Read length	Homopolymers
Proton (Thermo Fisher Scientific)	PR	10−15	200	4−24 h	149,000	Sequencing time Read length	Homopolymers
**Second-generation** **SMRT**							
PacBio RSII (Pacific Biosciences)	Fluo	0.5−1	60,000	>6 h	750,000	Sequencing time Read length	Price Many reading errors
Sequel (Pacific Biosciences)	Fluo	5−10	60,000	>20 h	350,000	Sequencing time Read length	Price Many reading errors
MinION (ONT)	IC	10−20	>100,000	0.5−48 h	1000	Sequencing time Read length Price Size	Many reading errors
GridION (ONT)	IC	50−100	>100,000	0.5−48 h	2400	Sequencing time Read length	Many reading errors
PromethION (ONT)	IC	480−960	>100,000	0.5−48 h	25,000	Sequencing time Read length	Many reading errors

**Table 6:** Comparison of different databases for the study of mutations in resistance genes to first- and second-line anti-TB drugs. AMC: Amoxicillin-clavulanic acid; AMK Amikacin; BDQ: Bedaquiline; CAP:Capreomycin; CLA: Clarithromycin; CLO: Clofazimine; CYC: Cycloserine; ETH: Ethambutol; ETHI Ethionamide; FQ Fluoroquinolones; ISO Isoniazid; KAN: Kanamycin; LIN: Linezolid; PAS Para-amino salicylic acid ; PRT: Prothionamide ; PYR: Pyrazinamide ; RFB: Rifabutine; RIF Rifampicin; STR Streptomycine; THI: Thiocetazone.

Database (Ref.)	Link	Sources and Update	Genes or Promoters Included	ATB Included				Phenotype/Genotype Correlation
**Tuberculosis Drug Resistance Mutation database (TBDReaMDB)** [29]	http://www.tbdreamdb.com/ (accessed on 31 May 2023)	Free access. Data from the literature. Includes mutations that are more often associated with resistance than with antibiotic susceptibility. Data on nucleotides and amino acids. Lack of information on update dates.	39	RIF ISO ETH PYR STR	AMK FQ ETHI PAS			Only isolates that have been characterised by phenotypic susceptibility testing are considered. The number of susceptible and resistant isolates carrying the mutation must be specified.
**Mutation BioInformatics Identification (MUBII-TB-DB)** [30]	https://umr5558-proka.univ-lyon1.fr/mubii/mubii-select.cgi (accessed on 31 May 2023)	Free access. Literature data (systematic review of MEDLINE-referenced publications before March 2013). Data on nucleotides and amino acids.	7	RIF ISO ETH	PYR FQ AMK			Only isolates that have been characterised by phenotypic susceptibility testing are considered.
**PhyResSe** [31]	http://phyresse.org (accessed on 31 May 2023)	Free access. Data from the literature and experiments in the laboratory. Possible input from users.	12	RIF ISO ETH PYR STR AMK	FQ ETHI PAS CAP KAN PRT			Links to the relevant studies are provided for each mutation.
**Tuberculosis Drug resistance Database (TBDR)** [32]	https://tbdr.lshtm.ac.uk/ (accessed on 31 May 2023)	Free access. Literature data from 2011 to 2014 and data from other databases. (TBDReaMDB and Broad/GTBDR). Data on nucleotides and amino acids.	29	RIF ISO ETH PYR STR	AMK FQ ETHI PAS	AMC CAP KAN CLA CYC	PRT RFB LIN THI	Phenotypic data per drug required, not just classification of isolates according to MDR or XDR criteria.
**Drug Resistance Associated Genes database (DRAGdb)** [33]	http://bicresources.jcbose.ac.in/ssaha4/drag/ (accessed on 31 May 2023)	Free access. Literature data, publications referenced in PubMed until March 2018. Data on nucleotides and amino acids.	12	RIF ISO ETH	PYR FQ STR			Comparison of isolates to phenotypic data is not mandatory.
**Resistance Sniffer** [34]	http://resistancesniffer.bi.up.ac.za/ (accessed on 31 May 2023)	Data from databases: GMTV (Genome Variation *Mycobacterium Tuberculosis* Variation), PATRIC (Pathosystems Resource Integration Center), and from a SAMRC (South African Medical Research Council) study.	23	RIF ISO ETH PYR	STR AMK FQ ETHI	PAS CAP KAN		Only isolates that have been characterized by phenotypic susceptibility tests are taken into account.
**Relational Sequencing Tuberculosis Data Platform (ReSeqTB)** [35]	https://platform.reseqtb.org (accessed on 31 May 2023)	Centralized repository for the continuous collection, active management, and validation of data.	/	/				Retrospective and prospective data with phenotypic sensitivity data as well as clinical data are taken into account.
**ResFinder 4.0** **And PointFinder** [36,37]	https://cge.food.dtu.dk/services/ResFinder/ (accessed on 31 May 2023)	Free access. Literature data reviewed and selected by scientists and communication with researchers. Data on nucleotides. Possibility of including your own database locally. Database not only dedicated to mycobacteria.	36	RIF ISO ETH	PYR STR AMK FQ	ETHI PAS CAP KAN	CYC LIN BDQ CLO	For each referenced mutation, the user obtains the PubMed ID of the article linking the genotype observed to the predicted resistance phenotype.
**Deeplex^®^ Myc-TB** [38]	https://deeplex.bluebee.com/deeplex/#!login (accessed on 31 May 2023)	Data from the literature and other databases: PhyResSe and ReSeqTB.	18	RIF ISO ETH PYR	STR AMK FQ ETHI	PAS CAP KAN CYC	LIN BDQ CLO	No information.

## Data Availability

Not applicable.
